# Targeting NIK-mediated microglial polarization: a novel therapeutic strategy for diabetic retinopathy

**DOI:** 10.1186/s12967-026-08728-4

**Published:** 2026-08-17

**Authors:** Hongxue Li, Qian Xu, Chengye Xu, Yuxin Hu, Hongyu Kuang

**Affiliations:** First Affiliated Hospital of Harbin Medical University, Harbin, China

## Abstract

**Background:**

Diabetic retinopathy (DR) is a sight-threatening microvascular complication characterized by blood-retina barrier (BRB) disruption. While inflammation is central to early DR, the role of the non-canonical NF-κB pathway remains poorly understood.

**Methods:**

We established a murine DR model and performed RNA-seq. Retinal microglial activation and the expression of NF-κB-inducing kinase (NIK) were assessed via immunohistochemistry and Western blot. In vitro, we employed AGEs-treated HMC3 microglia and a co-culture system with human retinal microvascular endothelial cells (hRMECs) under NIK knockdown or pharmacological inhibition (using the NIK inhibitor B022). Molecular mechanisms were delineated using ChIP-qPCR and pathway analysis.

**Results:**

RNA-seq revealed significant enrichment of non-canonical NF-κB signaling in DR. NIK was significantly upregulated in DR retinas, specifically in microglia. NIK knockdown in vitro attenuated microglial activation, suppressed pro-inflammatory M1 markers (iNOS, TNF-α, IL-1β, IL-6), and promoted anti-inflammatory M2 markers (Arg-1, IL-10). Mechanistically, NIK drives microglial polarization via the non-canonical NF-κB pathway (IKKα/p52). In co-culture, NIK knockdown or B022 treatment rescued hRMECs from AGEs-activated microglia-induced dysfunction, improving viability, reducing apoptosis, and restoring barrier integrity. Intravitreal administration of B022 in DR mice mitigated retinal vascular leakage, reduced apoptosis, and rebalanced M1/M2 polarization.

**Conclusions:**

Our study identifies NIK as a master regulator of microglial polarization in DR via non-canonical NF-κB signaling. By disrupting BRB integrity, NIK-driven neuroinflammation is a critical pathogenic mechanism. The NIK inhibitor B022 demonstrates strong therapeutic potential, positioning NIK as a novel molecular target for treating diabetic retinopathy.

**Supplementary Information:**

The online version contains supplementary material available at https://doi.org/10.1186/s12967-026-08728-4.

## Introduction

Diabetes mellitus, a metabolic disorder characterized by chronic hyperglycemia, continues to rise in global prevalence. Environmental toxins, psychological stress, and lifestyle factors are known contributors to its pathogenesis [1]. Diabetic retinopathy (DR), a major microvascular complication, affects approximately one-third of diabetic individuals and represents a leading cause of blindness and visual impairment among adults in many countries [2]. Vision loss significantly impairs patients’ quality of life and carries profound social and psychological consequences. Despite its clinical impact, effective treatments to prevent the onset or progression of DR remain limited.

Historically viewed as a microvasculopathy, DR involves vascular endothelial dysfunction as an early pathological event [3]. Severe endothelial impairment leads to blood-retina barrier (BRB) breakdown, vascular leakage, hemorrhage, retinal detachment, and eventual vision loss [4]. Growing evidence, however, indicates that inflammatory processes precede overt vasculopathy and play a critical role in driving retinal endothelial dysfunction in diabetes [5, 6]. Targeting inflammation may thus offer promising strategies for endothelial protection in DR.

As the resident immune cells of the retina and central nervous system, microglia are essential in maintaining retinal homeostasis [7, 8]. They modulate neuronal and vascular activity through the release of cytokines and neurotrophic factors and help preserve vascular integrity by monitoring microenvironmental changes [9] and communicating with endothelial cells [10]. Under pathological conditions, microglia become activated and undergo morphological changes, polarizing toward either a pro-inflammatory M1 phenotype or an anti-inflammatory M2 phenotype [11]. This dynamic polarization allows microglia to adapt to environmental cues, and disruption of the M1/M2 balance is now recognized as a key mechanism in DR-related neuroinflammation [12]. Nevertheless, the specific mechanisms through which microglial polarization contributes to endothelial dysfunction in DR are still not fully understood.

The NF-κB signaling pathway regulates diverse biological processes including inflammation, immunity, apoptosis, and cell proliferation. The canonical NF-κB pathway mediates IκB degradation, leading to nuclear translocation of the RelA: p50 dimer and subsequent gene transcription [13]. In contrast, the non-canonical pathway facilitates nuclear accumulation of the RelB: p52 heterodimer to regulate distinct target genes [14]. While the canonical NF-κB pathway is well-established in DR pathogenesis [15], the potential involvement of the non-canonical pathway remains unclear. NF-κB-inducing kinase (NIK) serves as a central regulator of non-canonical NF-κB activation [16]. Although best known for its roles in immune regulation, NIK has recently been implicated in metabolic diseases [17–19] and microglia-mediated central nervous system disorders [20]. Despite these associations, the role of NIK in diabetes-induced microglial polarization has not yet been elucidated.

In this study, we investigate the pathological function of NIK in DR. Our findings demonstrate that NIK activation promotes microglial polarization and vascular endothelial injury via the non-canonical NF-κB pathway. These results highlight the translational potential of targeting NIK and its downstream inflammatory signaling as a novel therapeutic strategy for the early prevention and treatment of diabetic retinopathy.

## Materials and methods

### Animals

C57BL/6 N male mice (6–8 weeks old, 23–30 g) were obtained from Jiangsu Huachuang Xinnuo Pharmaceutical Technology Co., Ltd. (Jiangsu, China). The animals were housed under controlled temperature (22 ± 2 °C) and humidity (40–70%) with a 12-hour light/dark cycle. After one week of acclimatization, the mice were randomly assigned to two groups: a control group (CON, *n* = 8) fed a standard diet, and a model group (*n* = 24) fed a high-fat diet. At the eighth week, diabetes was induced in the model group via intraperitoneal injection of streptozotocin (STZ; Sigma, USA) at 35 mg/kg, prepared as a 1% solution. Fasting blood glucose (FBG) was measured 72 h post-injection for two consecutive days using a glucometer. Mice with FBG ≥ 7.8 mmol/L were considered diabetic. The model group was further randomized into a diabetic retinopathy group (DR, *n* = 12) and a DR group treated with B022 (DR+B022, *n* = 12), a NIK small-molecule inhibitor (MCE, USA), administered via intravitreal injection at 30 mg/kg twice daily. Control and DR groups received intravitreal saline. After 12 weeks, mice were euthanized under anesthesia, and eyeballs were enucleated for analysis. All procedures were approved by the Ethics Committee of The First Affiliated Hospital of Harbin Medical University.

### RNA sequencing

Total RNA was extracted for library construction. mRNA was isolated using poly-T oligo-attached magnetic beads, followed by PCR amplification and purification with AMPure XP beads. Library quality was verified using an Agilent Bioanalyzer 2100 system. Sequencing was performed on the Illumina NovaSeq 6000 platform. Differential gene expression analysis was conducted using DESeq2 (v1.20.0) in R, with significance thresholds set at |Fold Change| > 1 and adjusted p-value < 0.05. Functional enrichment analyses for KEGG and Gene Ontology (GO) terms were performed using cluster Profiler. Gene Set Enrichment Analysis (GSEA v4.3.2) was used to identify enriched pathways.

### H&E staining

Eyeballs were fixed in 4% paraformaldehyde, dehydrated through graded ethanol and xylene, and embedded in paraffin. Section  (4 μm) were dewaxed, rehydrated, and stained with hematoxylin and eosin. After dehydration and clearing, sections were mounted with neutral gum and imaged under a Nikon microscope (Japan) at 400× magnification.

### Immunohistochemistry

Deparaffinized retinal sections underwent antigen retrieval in citric acid (pH 6.0), endogenous peroxidase blocking with 3% H₂O₂, and serum blocking. Primary antibodies against NIK (1:100; Bioss, China), TNF-α (1:400; Wanleibio, China), IL-1β (1:400; Wanleibio, China), IL-6 (1:400; Wanleibio, China), IL-10 (1:400; Wanleibio, China), cleaved-caspase 3 (1:400; Wanleibio, China), BAX (1:400; Wanleibio, China), and Bcl-2 (1:400; Wanleibio, China) were applied overnight at 4 °C. After PBS washing, sections were incubated with secondary antibodies, developed with DAB, and counterstained with hematoxylin. Images were captured at 400× magnification.

### Vascular permeability assay

Mice were anesthetized with isoflurane, and FITC-dextran (40 kDa; Sigma, USA) was perfused through the left ventricle. Retinas were dissected, flat-mounted, and imaged using a fluorescence microscope (EVOS FL Auto, Life Technologies, USA) to assess vascular leakage.

### Cell culture and treatment

HMC3 cells (Procell, China) were maintained in MEM supplemented with 10% FBS and 1% penicillin-streptomycin. Human retinal microvascular endothelial cells (hRMECs; Bena, China) were cultured in ECM containing 5% FBS, 1% penicillin-streptomycin, and endothelial cell growth supplement. RPE cells (Procell, China), RMC cells (Procell, China) and RGC cells (Procell, China) were maintained in DMEM supplemented with 10% FBS and 1% penicillin-streptomycin. All cells were kept at 37 °C in 5% CO₂. For experiments, HMC3 cells in logarithmic growth phase were seeded at 5 × 10⁵cells/well and treated as follows: Control, AGEs (200 µg/mL), and AGEs + B022 (10 µM pre-treatment for 6 h). After 24 h, cells and supernatants were collected.

### Co-culture system

HMC3 cells and hRMECs were co-cultured using Transwell inserts. HMC3 cells were seeded in apical chambers and hRMECs in bottom wells. After 24 h, HMC3 cells were treated with AGEs ± B022 for another 24 h. Cells and supernatants were harvested separately.

### Lentiviral transduction

Lentiviral vectors encoding NIK shRNA (pLV-U6-ZsGreenl-puro-shNIK) and negative control (pLV-CMV-ZsGreenl-puro-NC) were from JuRen Bioscience (China). HMC3 cells were transduced with lentivirus in the presence of polybrene (5 µg/mL) and selected with puromycin (Beyotime, China). Knockdown efficiency was confirmed by Western blot and RT-qPCR.

### Immunofluorescence

Retinal tissues or fixed/permeabilized HMC3 cells were blocked and incubated with primary antibodies against Iba-1(1:200; Bioss, China), CD68(1:200; Bioss, China), CD206 (1:200; Bioss, China), NIK (1:200; Bioss), or NFKB2 (1:50; Proteintech, USA), followed by Alexa Fluor-conjugated secondary antibodies and DAPI. Images were acquired using confocal or fluorescence microscopy.

### Flow cytometry

Apoptosis was assessed using Annexin V-FITC/PI staining (Elabscience, China). For polarization analysis, cells were stained with CD68 (Elab Fluor 488) and CD206 (FITC) antibodies (1:20; Elabscience). Data were collected on a BD Accuri C6 flow cytometer and analyzed with FlowJo (v10.8.1).

### ChIP-qPCR

Chromatin immunoprecipitation was performed using a BeyoChIP™ kit (Beyotime, China) with anti-NFKB2 antibody (Proteintech, USA). Precipitated DNA was analyzed by qPCR for INOS and ARG1 promoter enrichment.The primer sequences for the following real-time qPCR included: INOS forward: 5′-CTGGTGTCCCCCATTACCAC-3′, reverse: 5′-CTGAGAGGAGTGGGATGGGA-3′ ARG1 forward: 5′-AAGCTCGACGGTTAAGTGGA-3′, reverse: 5′-ACCATGTGTCCGATGCAGTT-3′.

### Western blot

Proteins were extracted using RIPA buffer with inhibitors, quantified by BCA assay, separated by SDS-PAGE, and transferred to PVDF membranes. After blocking, membranes were incubated with primary antibodies against NIK (1:200; Bioss), IKKα (1:1000; Wanleibio), p-IKKα (1:1000; CST), iNOS(1:1000; Proteintech), NFKB2(1:1000; Proteintech), Arg-1(1:1000; Proteintech), β-tubulin(1:1000; Proteintech), and β-actin (1:1000; Proteintech), followed by HRP-conjugated secondary antibodies. Blots were developed with ECL and analyzed using ImageJ.

### Cell viability assay

Cells were seeded in 96-well plates (1 × 10⁴cells/well) and treated as indicated. Viability was assessed using CellTiter-Glo reagent (Promega, USA) and measured with a microplate reader.

### TUNEL assay

Apoptosis in retinal sections was detected using a TUNEL kit (Elabscience, China) according to manufacturer instructions. Nuclei were counterstained with DAPI, and images were captured by fluorescence microscopy.

### Cell permeability assay

hRMECs and HMC3 cells were co-cultured in Transwell systems. FITC-dextran (70 kDa, 100 µg/mL) was added to the upper chamber, and fluorescence in the lower chamber was measured at 480/520 nm.

### ELISA

Cytokine levels (TNF-α, IL-1β, IL-6, IL-10) in vitreous humor or cell supernatants were quantified using ELISA kits (Jianglaibio, China) according to manufacturer protocols.

### Quantitative real-time PCR

RNA was extracted, reverse-transcribed, and amplified using SYBR Green Master Mix (YEASON, China) on an Applied Biosystems 7500 system. Gene expression was normalized to ACTIN and calculated via the 2^–^ΔΔCT^ method. Primer sequences: MAP3K14 forward: 5′-TGTGGGAAGTGGGAGATCCTA-3′ reverse: 5′-GGCTGAACTCTTGGCTATTCTCA-3′, ACTIN forward: 5′-GGGAAATCGTGCGTGACATT-3′ reverse: 5′- GGAACCGCTCATTGCCAAT-3′.

### Statistical analysis

Data are presented as mean ± SD. Analyses were performed using GraphPad Prism 8.0. Student’s t-test was used for comparisons, with *p* < 0.05 considered statistically significant.

## Results

### RNA sequencing of DR mice’s retina identifies novel vascular inflammation-associated pathway

**Fig. 1:**
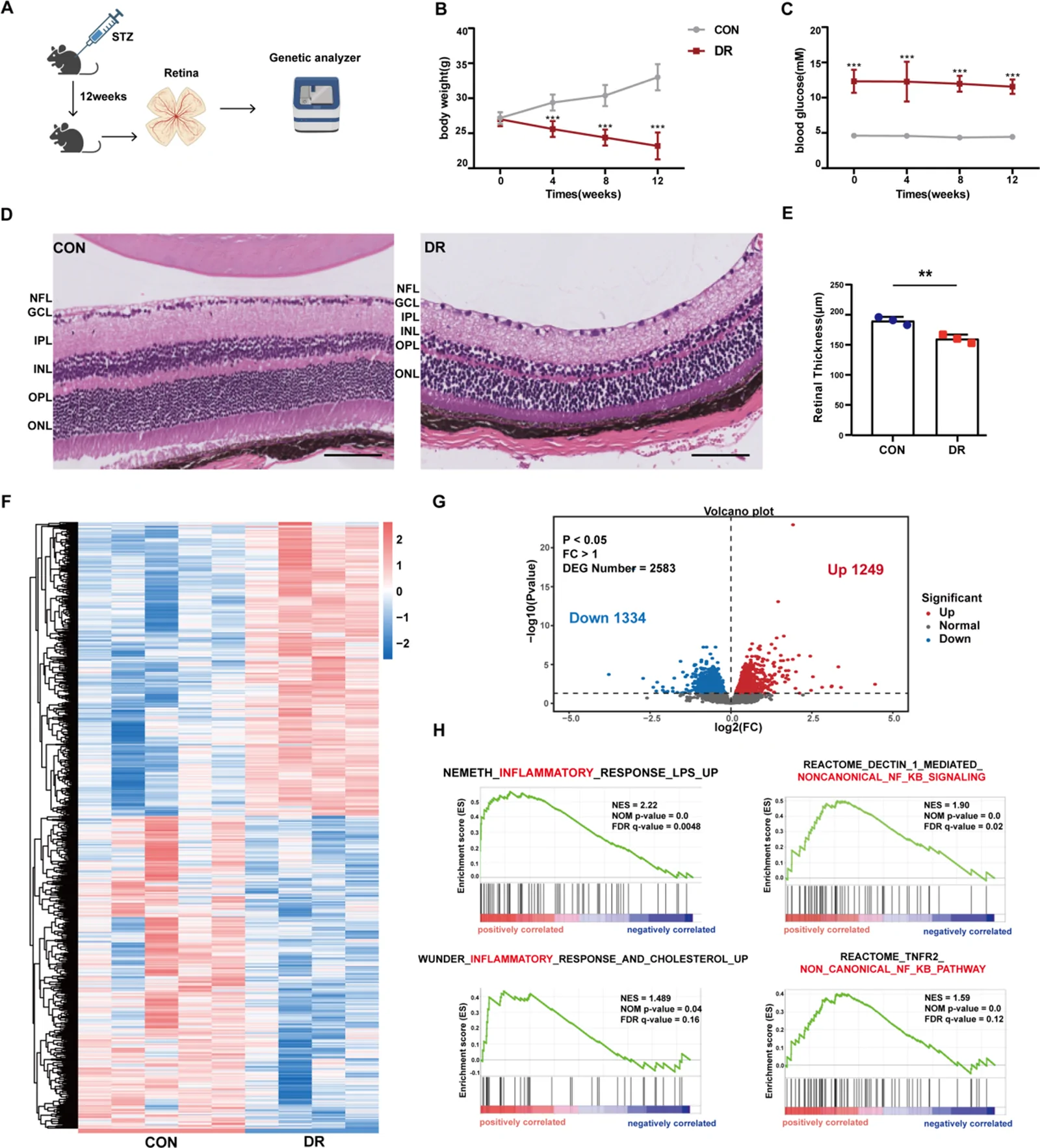
Established DR mice model and RNA-seq analysis of Mice retina. **A**. Animal study flowchart. **B**. The changes in body weight in mice with diabetes progression. **C**. The changes of fasting blood glucose in mice with diabetes progression. **D**. HE staining of mice retinal tissue. Scale bar: 100 μm. NFL: nerve fiber layer. GCL: ganglion cell layer. IPL: inner plexiform layer. INL: inner nuclear layer. OPL: outer plexiform layer. ONL: outer nuclear layer. **E**. retinal thickness analysis of mice retinal tissue. **F**. Heat map illustrated DEGs expression in Con group and DR group. A Fold Change > 1 and an adjusted P value < 0.05. Color bar, log2(FC). **G**. Volcano map illustrated DEGs expression in Con group and DR group. H. Pathways related to DR were positive correlated, as revealed by GSEA. NES, normalized enrichment score; FDR, false discovery rate. Data are presented as means ± SD(*n* = 3). **P < 0.01. ***P < 0.001

The diabetic mice model was established by intraperitoneal injection of STZ combined with high-fat feeding. After diabetes onset, over 12 weeks, the body weight of the non-diabetic control mice increased with time. However, the body weight of the diabetic mice decreased significantly (Fig. 1B). Blood glucose levels in the diabetic group increased, especially compared with the control condition (Fig. 1C). The elevated blood glucose level and the decreased body weight compared with standard control indicated the successful establishment of the diabetic mice model.

We performed HE staining for pathological sections of the mice’s retina in the 12th week to observe the condition of diabetic retinopathy. The retina of non-diabetic mice had normal morphology and structure, with cells grouped neatly and closely, and the system between cells in each layer was neat, as revealed by the microscopic structure. Diabetic mice had a slightly disorganized distribution of the retinal structure, and the thickness shrank noticeably. The number of cells in each layer was drastically reduced, their organization became disorganized, and the distance between cells grew noticeably (Fig. 1D-E). Our findings are consistent with previous research, indicating that our DR mouse model has been successfully established.

To further find the mechanism in DR, we conducted RNA-seq analysis in DR mice retina. In comparison to the Control group, DR group have significant down-regulation of 1334 genes and up-regulation of 1249 genes (Fig. 1F-G). Next, we analyzed the DR-related signaling pathways in GSEA, the subsequent pathways exhibited significant enrichment: NEMETH_INFLAMMATORY_RESPONSE_LPS_UP, DECTIN_1_MEDIATED_NONCANONICAL_NF_KB_SIGNALING, WUNDER_INFLAMMATORY_RESPONSE_AND_CHOLESTEROL_UP, REACTOME_TNFR2_NON_CANONICAL_NF_KB_PATHWAY (Fig. 1H). We found there are many inflammation related pathway, notably the non-canonical NF-κB pathway, may serve as valuable sources of insight into DR development, similar to the canonical NF-κB pathway.

### NIK is overexpressed in DR mice’s retina and microglia

**Fig. 2:**
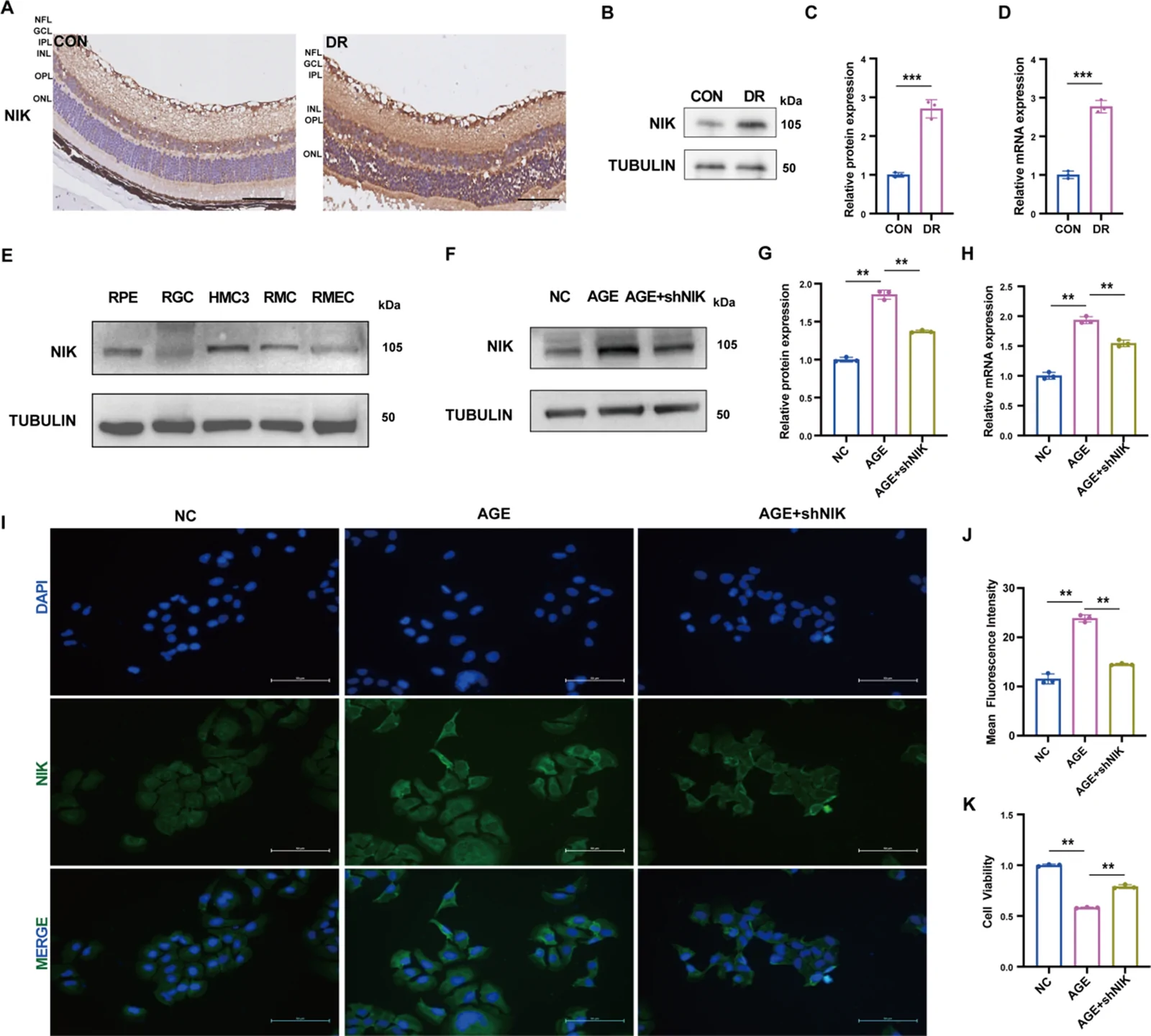
NIK is Highly expressed in diabetic mice retina and AGEs-stimulated HMC3 cells. (**A**) The expression of NIK in retina was detected by IHC. Scale bar = 50 μm. (**B**) The expression of NIK in retinas was detected by WB. (**C**) Relative protein expression of NIK in retina. (**D**) Relative mRNA expression of NIK in retina. (**E**) The expression of NIK in different cell line were detected by WB. (**F**) Determination of NIK expression in HMC3s by WB. (**G**) Relative protein expression of NIK in HMC3. (**H**) Relative mRNA expression of NIK in HMC3. (**I**) The expression of NIK in HMC3 was detected by IF. Scale bar = 50 μm. (**J**) Mean fluorescence intensity of NIK. K. Cell viability assay of the HMC3. NFL: nerve fiber layer. GCL: ganglion cell layer. IPL: inner plexiform layer. INL: inner nuclear layer. OPL: outer plexiform layer. ONL: outer nuclear layer. Data are presented as means ± SD(*n* = 3). **P < 0.01, ***P < 0.001

NIK is the core molecule of the non-canonical NF-κB pathway. We first examined NIK expression in the retina of DR mice to assess the function of NIK in DR. According to the immunohistochemical analysis (Fig. 2A), WB (Fig. 2B-C) and RT-qPCR (Fig. 2D) results, NIK was diffusely expressed across every layer of the mice’s retina. The expression of NIK was considerably increased in each layer of the DR mouse retina when compared to non-diabetic mice, particularly in the ganglion cell layer (GCL), inner plexus layer (INL), and outer plexus layer (OPL). These results prove that NIK is activated in the retina of DR mice.

Given the cellular heterogeneity of the retina, we aimed to identify the specific cell type in which NIK is expressed and mediates its function in DR development. Therefore, we performed WB (Fig. 2E) on cell types commonly implicated in DR: retinal pigment epithelial (RPE) cells, ganglion cells (RGC), microglia (HMC3), Müller cells (RMC) and endothelial cells (RMEC). The results demonstrated that NIK is significantly upregulated in HMC3 cells (a human microglial cell line), suggesting microglia may be the key cell type through which NIK contributes to DR pathogenesis. And then the subsequent in vitro experiments were performed in HMC3 cells.

Next, we established an in vitro cellular model with NIK knockdown. Using Advanced Glycation End products (AGEs) to simulate DR conditions in vitro, we assessed NIK knockdown efficiency via WB (Fig. 2F-G), RT-qPCR (Fig. 2H), and IF (Fig. 2I-J), alongside cell viability assays (Fig. 2K). Following AGEs intervention, NIK expression was markedly increased, accompanied by a significant reduction in cell viability. In contrast, the NIK knockdown group exhibited decreased NIK expression and restored cell viability.

### NIK regulates microglia M1/M2 polarization and RMECs dysfunction

**Fig. 3:**
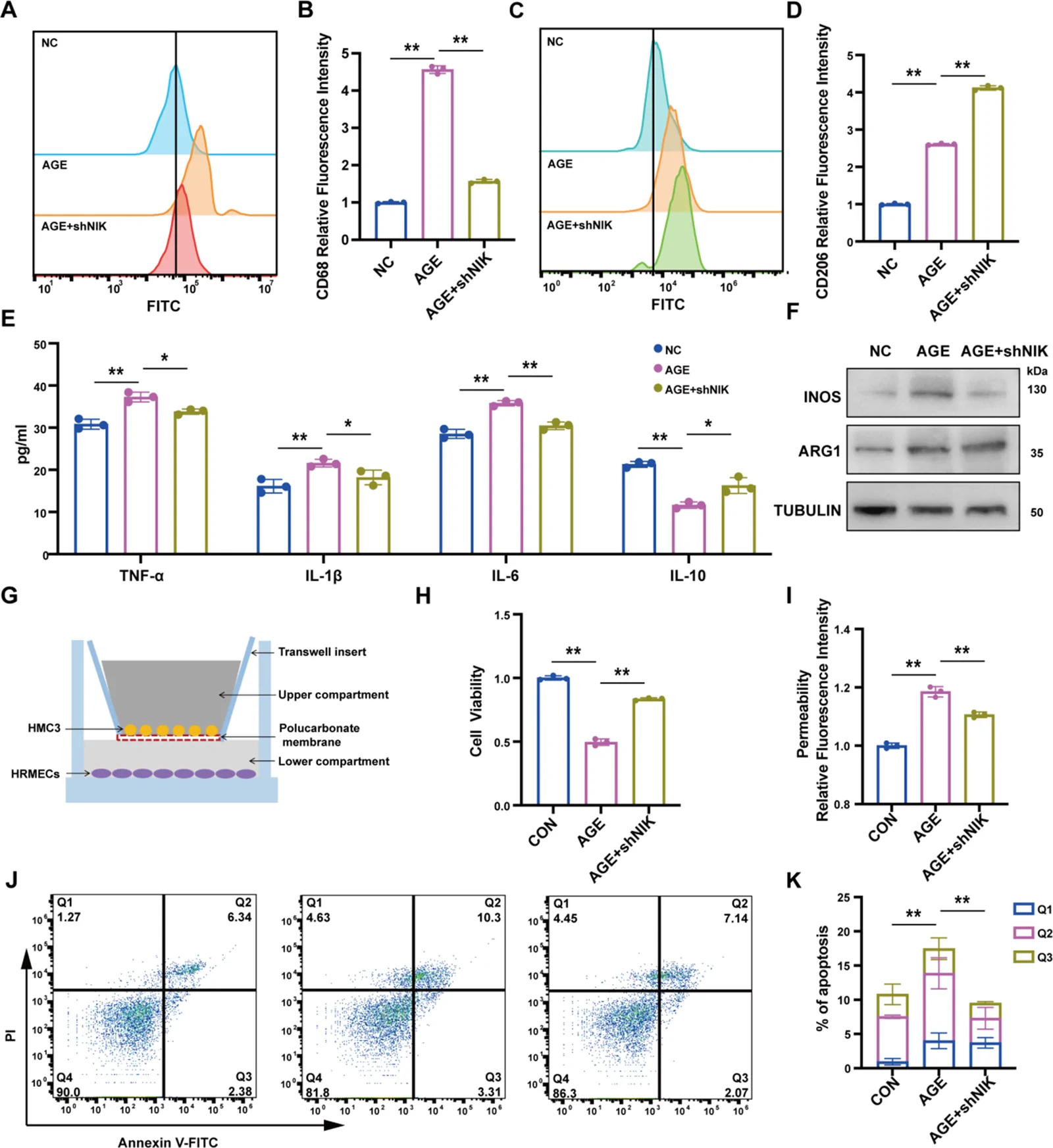
NIK regulate M1/M2 polarization-mediated apoptosis of hRMECs and BRB breakdown. **A**-**B**. CD68 antibody was used to stain cells for Flow Cytometry analysis. **C**-**D**. CD206 antibody was used to stain cells for Flow Cytometry analysis. **E**. The inflammatory factors in the supernatant were detected by ELISA assays. **F**. The expression of INOS and ARG1 were evaluated by WB. **G**. Co-culture system. **H**. Cell viability assay of the hRMECs. **I**. Relative fluorescence intensity detected by FITC-dextran. **J**-**K**. Cell apoptosis assay was performed with Annexin V/PI staining kits and analyzed by Flow Cytometry. Data are presented as means ± SD. *P < 0.05, **P < 0.01

To further investigate the role of NIK in DR, we assessed its effects on microglia M1/M2 polarization, inflammatory mediator secretion, and co-cultured RMEC barrier function following NIK knockdown in HMC3 microglia. Flow analysis revealed abundant CD68⁺ M1 and CD206⁺ M2 microglia in the AGE group, while NIK deficiency diminished the CD68⁺ population and augmented the CD206⁺ population (Fig. 3A-D). WB analysis corroborated this shift: iNOS expression increased and ARG1 decreased in the AGE group, whereas NIK knockdown attenuated iNOS but elevated ARG1 (Fig. 3F), and protein quantification is shown in the Fig.S1. ELISA of HMC3 supernatants demonstrated significantly elevated pro-inflammatory cytokines (TNF-α, IL-1β, IL-6) and decreased anti-inflammatory IL-10 in the AGE group versus controls (Fig. 3E). Strikingly, this inflammatory profile was reversed in the AGE+shNIK group, collectively establishing NIK’s essential role in establishing a pro-inflammatory milieu post-microglial polarization.

We next employed RMECs as blood-retinal barrier (BRB) models in co-culture with HMC3 microglia. Exposure to the HMC3-derived inflammatory milieu significantly reduced RMEC viability (Fig. 3H), increased barrier permeability (Fig. 3I), and enhanced apoptosis (Fig. 3J-K). Conversely, NIK knockdown restored viability, decreased apoptosis, and reduced permeability. These findings identify NIK-mediated microglial polarization as a key mechanism driving endothelial barrier dysfunction in DR.

### NIK regulates microglia M1/M2 polarization via the non-canonical NF-κB pathway

**Fig. 4:**
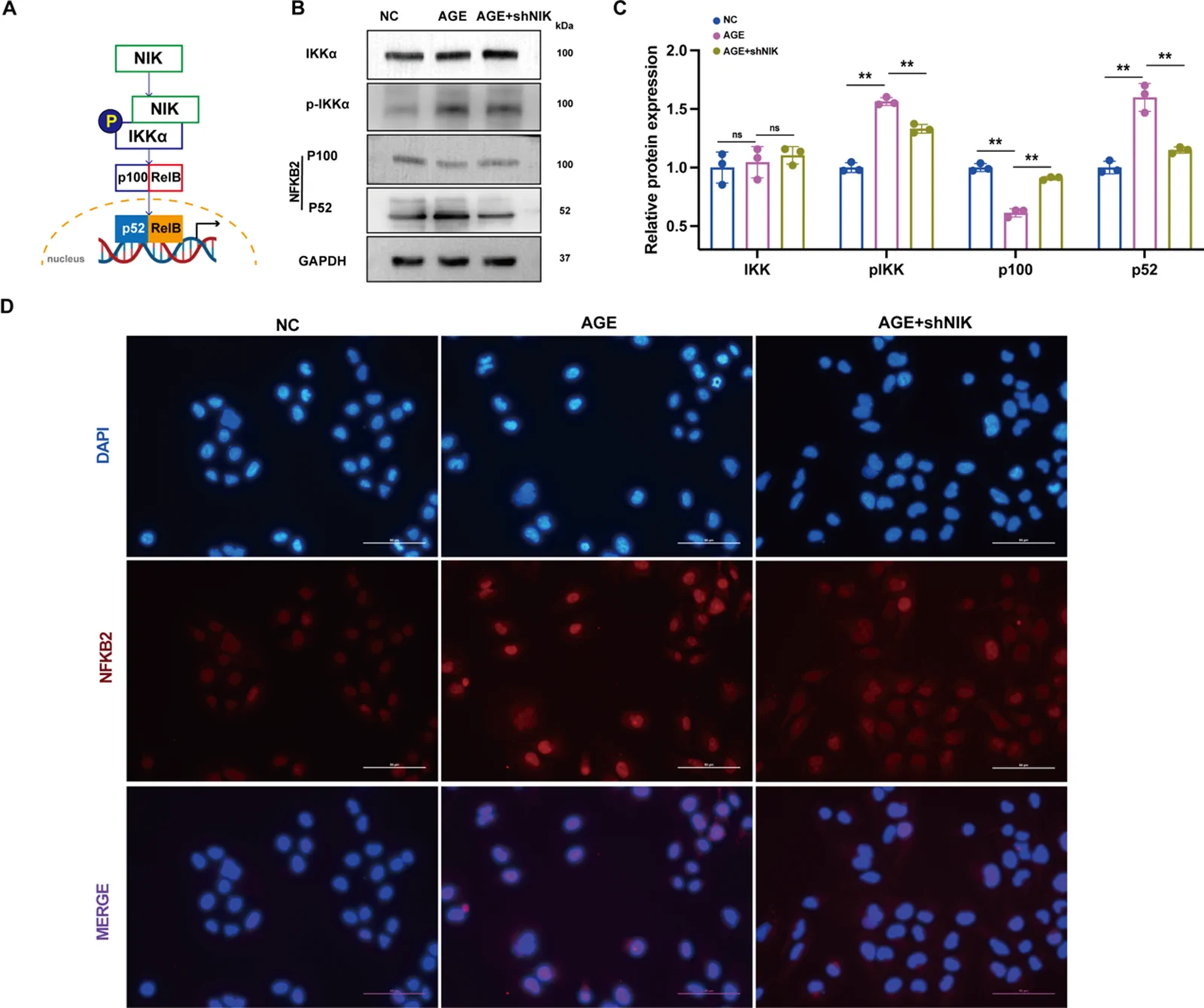
NIK regulates M1/M2 polarization through the non-canonical NF-κB pathway. **A** The non-canonical NF-κB pathway. **B**. The protein expression of IKKα, p-IKKα, p100 and p52 were evaluated by WB. **C**. Relative protein expression of IKKα, p-IKKα, p100 and p52. **D**. The expression of NFKB2 was detected by IF. Scale bar = 50 μm. Data are presented as means ± SD. **P < 0.01

NIK is the core molecule of the non-canonical NF-κB pathway [21]. After external stimulation activates NIK in the cytoplasm, it phosphorylates the downstream protein IKK, which phosphorylates p100, causing it to degrade to p52, which is transferred to the nucleus and acts as a transcription factor with RelB to regulate cellular functions [22] (Fig. 4A). The expression of intracellular non-canonical NF-κB pathway was detected by WB. Results showed that the expression of p-IKKα and p52 were higher, and p100 was lower in the AGE group than the NC group (Fig. 4B-C). To further confirm nuclear translocation of p52, we assessed nuclear NFKB2 levels using immunofluorescence (IF) (Fig. 4D-E). Results demonstrated significantly higher NFKB2 expression within the nucleus in the AGEs group compared to controls. Collectively, these findings demonstrate that AGEs activate the non-canonical NF-κB pathway, which contributes to microglial M1/M2 polarization imbalance.

### B022 attenuates microglial M1/M2 polarization and reduces RMECs dysfunction

**Fig. 5:**
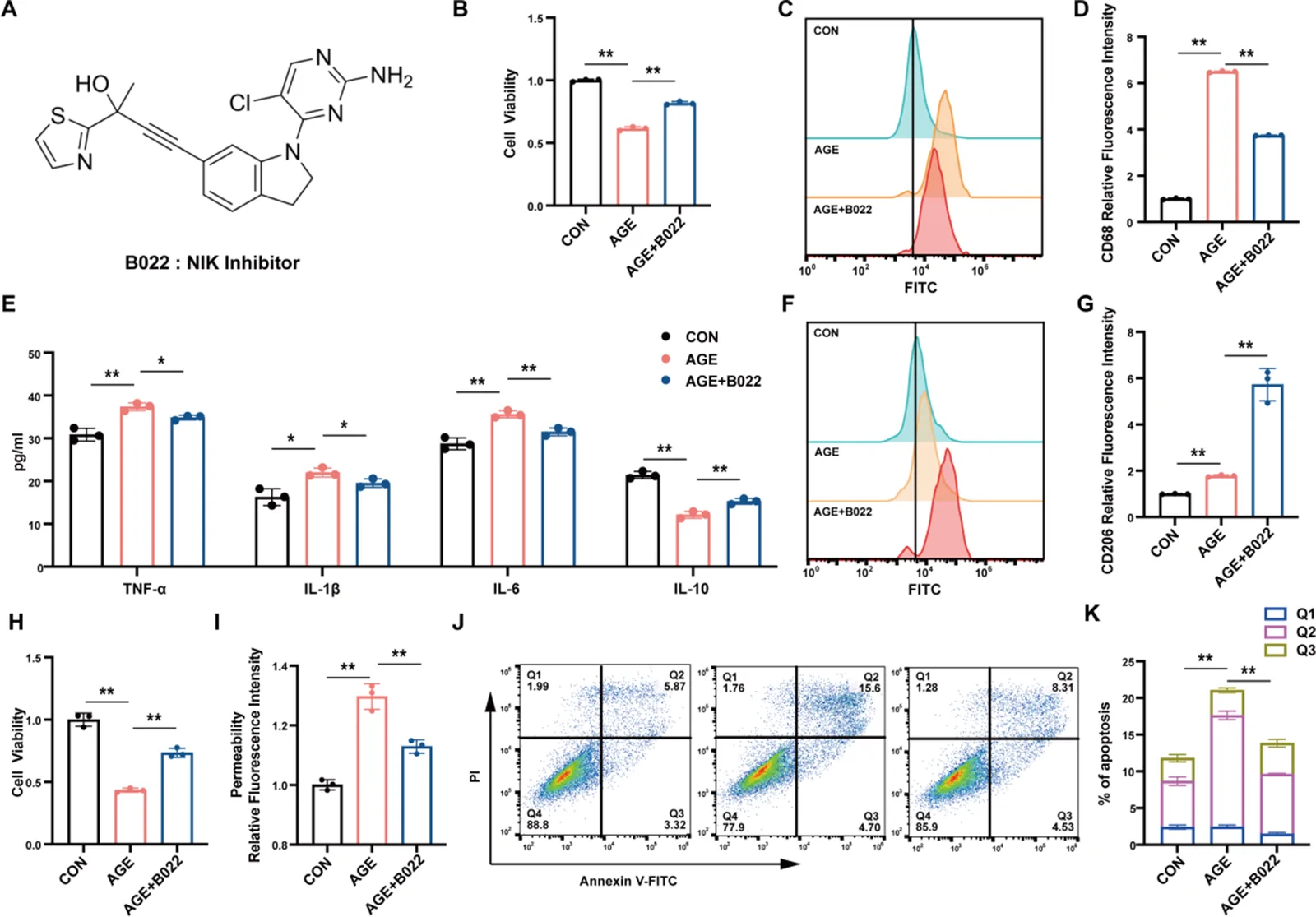
B022 attenuates microglial M1/M2 polarization and reduces RMECs dysfunction. **A** Chemical Structure of B022. **B**. Cell viability assay of the HMC3. **C**-**D**. CD68 antibody was used to stain cells for Flow Cytometry analysis. **E**. The inflammatory factors in the supernatant were detected by ELISA assays. **F**-**G**. CD206 antibody was used to stain cells for Flow Cytometry analysis. **H**. Cell viability assay of the RMECs. **I**. Relative fluorescence intensity detected by FITC-dextran. **J**-**K**. Cell apoptosis assay was performed with Annexin V/PI staining kits and analyzed by Flow Cytometry. Data are presented as means ± SD. *P < 0.05, **P < 0.01

Next, we treated HMC3 cells with the NIK inhibitor B022 to inhibit NIK activity. Cell viability assays confirmed that B022 significantly improved cell viability. Flow cytometry analysis revealed an increased abundance of CD68⁺ M1 and CD206⁺ M2 microglia in the AGE group. However, B022 treatment reduced the CD68⁺ population (Fig. 5C-D) and increased the CD206⁺ population (Fig. 5F-G). ELISA results indicated that B022 reversed the inflammatory profile. Furthermore, B022 also restored the viability of HMC3 co-cultured RMECs (Fig. 5H), reduced permeability (Fig. 5I), and decreased apoptosis (Fig. 5J-K). These results demonstrate that the small-molecule NIK inhibitor B022 attenuates AGE-induced microglial polarization and RMECs dysfunction.

### B022 reduce retina apoptosis and BRB breakdown in DR mice

**Fig. 6:**
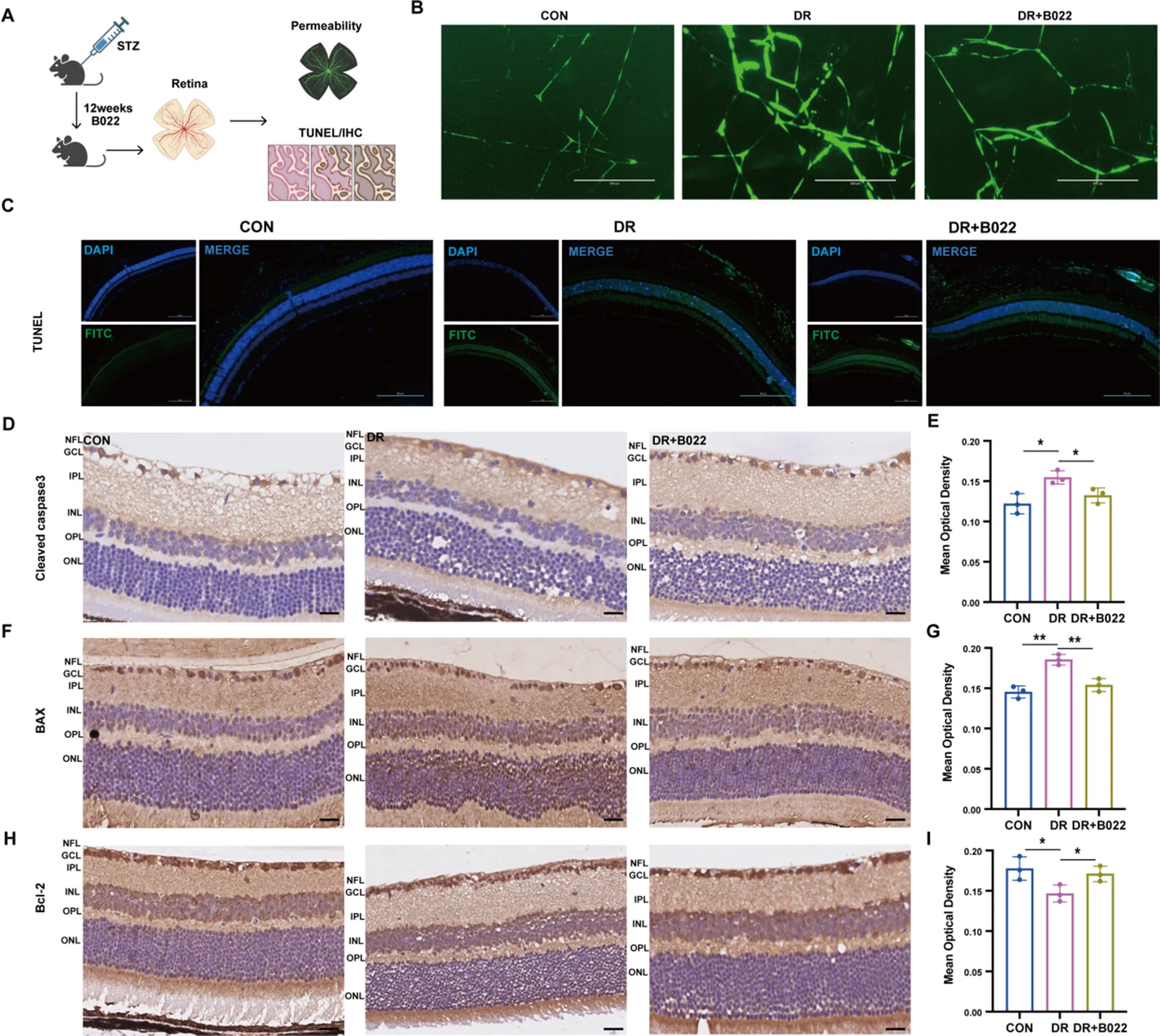
B022 reduce retina apoptosis and BRB breakdown in DR mice. (**A**) Animal study flowchart. (**B**) Vascular permeability was detected by FITC-dextran. Scale bar = 200 μm. (**C**) The apoptosis of retinal cells were detected by TUNEL assay. Scale bar = 50 μm. **D**-**E**. The expression of retinal apoptosis proteins Cleaved-caspase3 were detected by IHC. Scale bar = 50 μm. **F**-**G**. The expression of retinal apoptosis proteins BAX were detected by IHC. Scale bar = 50 μm. **H**-**I**. The expression of retinal apoptosis proteins BCL-2 were detected by IHC. Scale bar = 50 μm. NFL: nerve fiber layer. GCL: ganglion cell layer. IPL: inner plexiform layer. INL: inner nuclear layer. OPL: outer plexiform layer. ONL: outer nuclear layer. Data are presented as means ± SD(*n* = 3). *P < 0.05, **P < 0.01

To further clarify the effect of B022 on BRB, we used FITC-dextran to examine the vascular permeability. Compared with the Con group, Vascular leakage increased significantly in the DR group and improved in the DR+B022 group (Fig. 6B). Next, We used TUNEL assay and immunohistochemistry to examine the apoptosis of the mice retina and the expression of the TUNEL positive cells (Fig. 6C), pro-apoptotic proteins cleaved-caspase3 (Fig. 6D-E), BAX (Fig. 6F-G), and anti-apoptotic protein Bcl-2 (Fig. 6H-I) to confirm the impact of B022 on the diabetic retina. The above results suggest that B022 may be a new target in diabetic-mediated retinal BRB breakdown and cell apoptosis.

### B022 reduce microglial M1/M2 polarization and inflammatory microenvironment in the retina of DR mice

**Fig. 7:**
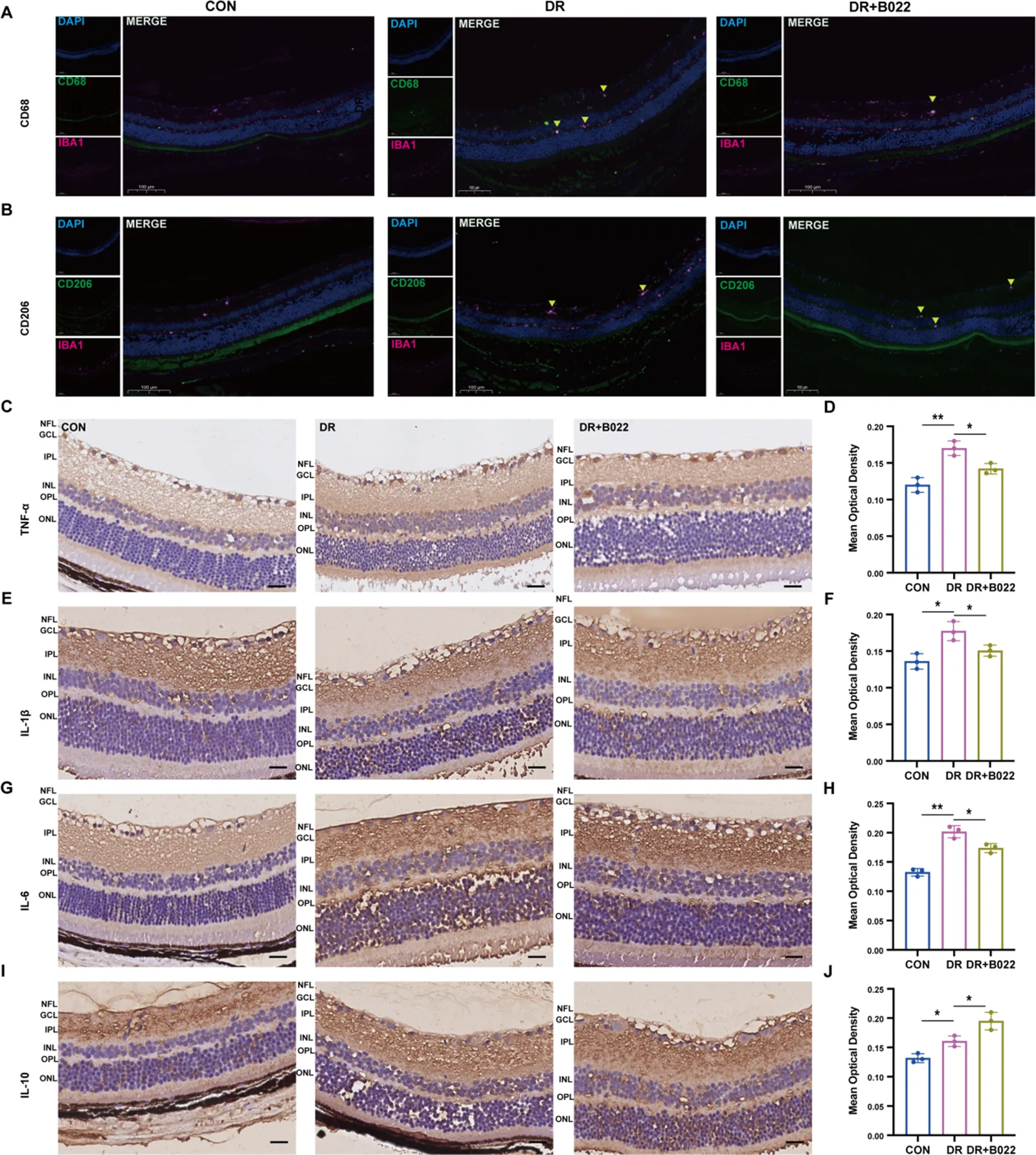
B022 reduce microglia M1/M2 polarization and inflammatory microenvironment in the retina of DR mice. **A** Retinal microglia were double stained with IBA1(green) and CD68(red). **B**. Retinal microglia were double stained with IBA1(green) and CD206(red). Scale bar = 100 μm. **C**-**D**. The expression of retinal inflammatory cytokines TNF-α were detected by IHC. Scale bar = 50 μm. **E**-**F**. The expression of retinal inflammatory cytokines IL-1β were detected by IHC. Scale bar = 50 μm. **G**-**H**. The expression of retinal inflammatory cytokines IL-6 were detected by IHC. Scale bar = 50 μm. **I**-**J**. The expression of retinal inflammatory cytokines IL-10 were detected by IHC. Scale bar = 50 μm. NFL: nerve fiber layer. GCL: ganglion cell layer. IPL: inner plexiform layer. INL: inner nuclear layer. OPL: outer plexiform layer. ONL: outer nuclear layer. Data are presented as means ± SD(*n* = 3). *P < 0.05, **P < 0.01

We compared the polarization of microglia in DR group and DR+B022 group using immunofluorescence, immunohistochemistry and WB to understand better the part played by B022. According to the immunofluorescence results (Fig. 7A-B), M1-type retinal microglia labeled with CD68^+^ and M2-type retinal microglia marked with CD206^+^ increased in the DR group compared to the control group, which was substantially different in the DR+B022 group. Consistently, quantitative Western blot analysis of retinal lysates revealed that iNOS/Arg1 ratio was significantly upregulated in the DR group compared to controls, and decreased in the DR+B022 group (Supplementary Fig. S1). Similarly, In the DR group, pro-inflammatory cytokines TNF-α (Fig. 7C-D), IL-1β (Fig. 7E-F) and IL-6 (Fig. 7G-H) and anti-inflammatory cytokine IL-10 (Fig. 7I-J) were higher expressions of the Con group. In the DR+B022 group, M1-type pro-inflammatory factors were significantly reduced, and M2-type anti-inflammatory factors were increased considerably, indicating that in the diabetic state, an inflammatory microenvironment caused by microglia polarization exists in the retina and that B022 improves the inflammatory environment in the retina. The above results suggest that the diabetic retina has an inflammatory microenvironment brought on by microglia polarization and B022 can reduce this inflammatory environment.

## Discussion

Diabetic retinopathy (DR) is a microvascular complication of diabetes. The retinal microvasculature consists of multiple cell populations that function in a coordinated manner [23]. The blood–retinal barrier (BRB), which tightly regulates the intraretinal environment, is composed of neuronal and glial cells interacting with pericytes [24]. Endothelial cells form the structural basis of the BRB, and inflammatory damage to these cells can increase microvascular permeability, leading to severe pathological outcomes such as retinal hemorrhage and detachment [25]. As a central initiator of the non-canonical NF-κB pathway, NIK (NF-κB-inducing kinase) modulates inflammatory responses across diverse cell types. Elevated NIK expression has been associated with metabolic disorders and inflammatory conditions [19]; however, its specific role in mediating inflammatory injury to the retinal vascular endothelium remains unclear. Elucidating the contribution of NIK in DR is therefore essential. B022, a competitive ATP inhibitor, has been shown to selectively target NIK and suppress NIK-driven hepatic inflammation in a dose-dependent manner [26]. In this study, we administered B022 via intravitreal injection in a mouse model of diabetic retinopathy. Our results demonstrate that B022 treatment reduces vascular leakage and attenuates retinal apoptosis. These findings suggest that inhibition of NIK enhances BRB integrity and suppresses apoptotic cell death in the diabetic retina.

Studies have shown that NIK promotes microglia activation and maintains CNS inflammation [20], Microglia are essential mediators of neuroinflammation. They are parenchymal macrophages in the brain and spinal cord that makeup 10–15% of the glial cell population. They constantly investigate the microenvironment and produce factors that impact peripheral neurons [27]. As a continuation of the CNS retinal microglia, the retina is also a critical therapeutic target for preventing and treating retinal neurodegenerative diseases [28]. In the retinal microenvironment of DR, some retinal cells can produce many inflammatory factors. Still, as an inflammatory leader, microglia can constantly monitor changes in their surroundings and maintain balanced functions by communicating with other retinal cells [12, 29, 30]. Upon encountering different stimulation, microglia transform from a surveillance state to an activated state to exhibit various tasks in the retina, transforming into M1 pro-inflammatory and M2 anti-inflammatory phenotypes [31], respectively. After activating, M1 phenotypes mainly secrete pro-inflammatory cytokines such as tumor necrosis factor (TNF-α), IL (interleukin)-1β, IL-6, and phagocytic oxidase-like iNOS. By contrast, M2 has a strong anti-inflammatory signature, expressing IL-10, Arg-1 [32]. The distinction between M1 and M2 microglia is fuzzy; these cells can constantly change their phenotype to accommodate changing conditions [33]. Hence, knowing the processes and signaling pathways that control microglial polarization may help identify new drug targets for disorders caused by neuroinflammation. Microglia hyperactivation and subsequent inflammation are thought to be linked to the progression of retinal diseases like age-related macular degeneration, macular degeneration, and diabetic retinopathy [34]. Our data indicated that the unbalance of M1/M2 microglia in C57BL/6 N mice with DR. This is consistent with a recent study in db/db mice [35], means M1/M2 polarization of microglia is linked to DR. Our results show that both M1 and M2 types of microglia were polarized in the retina of DR mice, but M1 types were somewhat more prevalent than M2 types, which resulted in higher amounts of pro-inflammatory factors: TNF-α, IL-1β, IL-6 in the retina. Admittedly, one limitation of this study is the lack of retinal flat-mount morphological analysis of microglia. To compensate, we performed quantitative Western blot analysis of M1 (iNOS) and M2 (Arg1) markers using remaining retinal lysates, which further supported our polarization findings. Future studies employing flat-mount techniques are warranted to validate the morphological changes. The development of DR has been strongly linked to the classical pro-inflammatory factors TNF-α, IL-1β, and IL-6, which can cause abnormal intercellular adhesion factors, trigger the inflammatory cycle by activating retinal microglia in response to their release from microglia, raise leukocyte adhesion levels, and release cytotoxic substances. Our findings suggest that the retina of DR mice develops an inflammatory microenvironment. After NIK inhibition, the B022 + DR group demonstrated a significant decrease in M1 microglia and an increase in M2 microglia, as well as a considerable suppression of the pro-inflammatory state and a substantial rise in anti-inflammatory factors, all of which pointed to a change in the retina’s inflammatory microenvironment.

Patients with diabetes have increased glucose in the circulation, which can accumulate in the retina, increase superoxide production in the mitochondria, and activate the polyol, protein kinase C, hexosamine, and renin-angiotensin pathways [36]. Metabolites such as advanced glycosylation end products (AGEs), ROS, glutathione, and lipid peroxides accumulate in the retina [37, 38]. AGEs are a significant stressor during diabetes due to the non-enzymatic glycosylation reaction of high glucose with various proteins, which can activate inflammatory responses in microglia cells, releasing a cascade of inflammatory mediators and creating a localized inflammatory microenvironment [39]. So, we developed a cell model of DR by induction of AGEs to HMC3 cells. HMC3 cell is a primary human fetal brain-derived microglia culture from CNS. There is no microglia cell line from the retina, so HMC3 was most commonly used in retinal diseases [40]. We discovered that AGEs drastically decreased the viability of HMC3 cells and caused M1/M2 polarization, releasing pro-inflammatory cytokines TNF-α, IL-1β, IL-6, and anti-inflammatory factor IL-10. Consistent with animal studies, we found that B022 combined with AGEs intervention significantly increased microglia viability and altered M1/M2 polarization and that inhibiting NIK expression decreased the release of M1 pro-inflammatory cytokines and increased the release of M2 anti-inflammatory cytokines. Hence, we propose that NIK expression may be one of the significant factors influencing the control of the inflammatory microenvironment in diabetic retinopathy.

Over-activated microglia can induce numerous impairments to the retinal vascular endothelium. Several recent studies have shown that microglia promote endothelial dysfunction by secreting different inflammatory agents, chemokines, and other cytokines [7, 41, 42] in addition to entering the vascular basement membrane and phagocytosing endothelial cells [10]. In the present study, we propose that NIK-mediated microglia polarization is a significant factor in endothelial dysfunction, so we developed an vitro blood-brain barrier model by co-culturing HMC3 cells with hRMECs. As we speculated, when hRMECs were co-cultured with AGEs and HMC3, the cell viability decreased significantly, and the number of apoptotic cells increased significantly. However, after the B022 treatment, the cell viability was restored, and the number of apoptotic cells was significantly decreased. the use of FITC to measure endothelial permeability was likewise in agreement with the previous information. The results indicate that NIK regulates retinal microglia M1/M2 polarization, forming an inflammatory microenvironment and inducing endothelial barrier dysfunction.

Nuclear factor kappa B (NF-κB) refers to a family of evolutionarily conserved transcription factors that regulate a wide range of biological processes [43]. In mammals, there are five NF-κB family members: RelA (p65), RelB, c-Rel, NF-κB1 (p105/p50) and NF-κB2 (p100/p52). NF-κB signaling transduction can be divided into canonical or non-canonical NF-κB signaling pathways, and is often triggered by inflammatory, pathogenic or stress signals [44]. The canonical NF-κB pathway is considered a central regulator of the inflammatory response in normal physiology, apidly and transiently, and has been well-studied to play an important role in DR [45]. However, the non-canonical pathway, which has only recently gained attention, regulate a strict and complex manner in human disease. In recent years, numerous new regulators of the non-canonical NF-κB signaling pathway have been identified, and abnormal activation of the pathway has been seen in a variety of disease settings [14]. Since NF-κB pathways are intertwined, whether non-canonical NF-κB pathways are also involved in the development of DR has not been explored. In the non-canonical NF-κB pathway, NIK activation results in the phosphorylation of IKKα and p100. This subsequently triggers partial proteasome processing to produce p52, a transcription factor that binds to RELB as a heterodimer in the nucleus [46]. It has been reported that in tumer associated macrophages, activation of this non-canonical NF-κB pathway predominates the conversion of the M1-like cells to the M2-like cells [47]. Therefore, we speculate that this passway may also be implicated in the polarization of retinal microglia. Lentiviruses harboring a short hairpin RNA targeting NIK was used to knockdown NIK in vitro. The results showed that M1-type microglia polarization was inhibited after NIK knockdown. We further explored if the effect of NIK regulates microglia polarization through a non-canonical NF-κB pathway, and it came out that the overall intracellular p-IKKα/p52 changed with NIK knockdown. The p-IKKα/p52 induced by AGEs was significantly reduced after NIK knockdown, while the p52 level in the nucleus was reduced. As an NF-κB subunit, p52 also functions as a transcription factor in the nucleus, regulating cellular regulation through binding to downstream target genes cellular senescence [48]. We found that AGEs can promote the transfer of p52 from the cytoplasm to the nucleus, which may enable it to function as a transcription factor to initiate downstream gene expression. In summary, we found that NIK can regulate microglia polarization by promoting p52 entry into the nucleus through activating non-canonical NF-κB signaling, which provides a new possible mechanism to slow down DR progression.

Currently, diabetes is one of the most important life-long progressive chronic diseases. As a serious-threatening complication of diabetes, DR is the leading cause of visual impairment and blindness worldwide [2]. Several different therapeutic strategies were performed in the treatment of diabetic retinopathy, such as steroid treatment, vitrectomy, laser, and anti-VEGF drug treatment [49]. However, these treatments have some drawbacks, including heavy economic burden, frequent injections, resistance to therapy in some patients, and a need for more specificity to address the underlying cause [3, 50].

## Conclusion

**Fig. 8:**
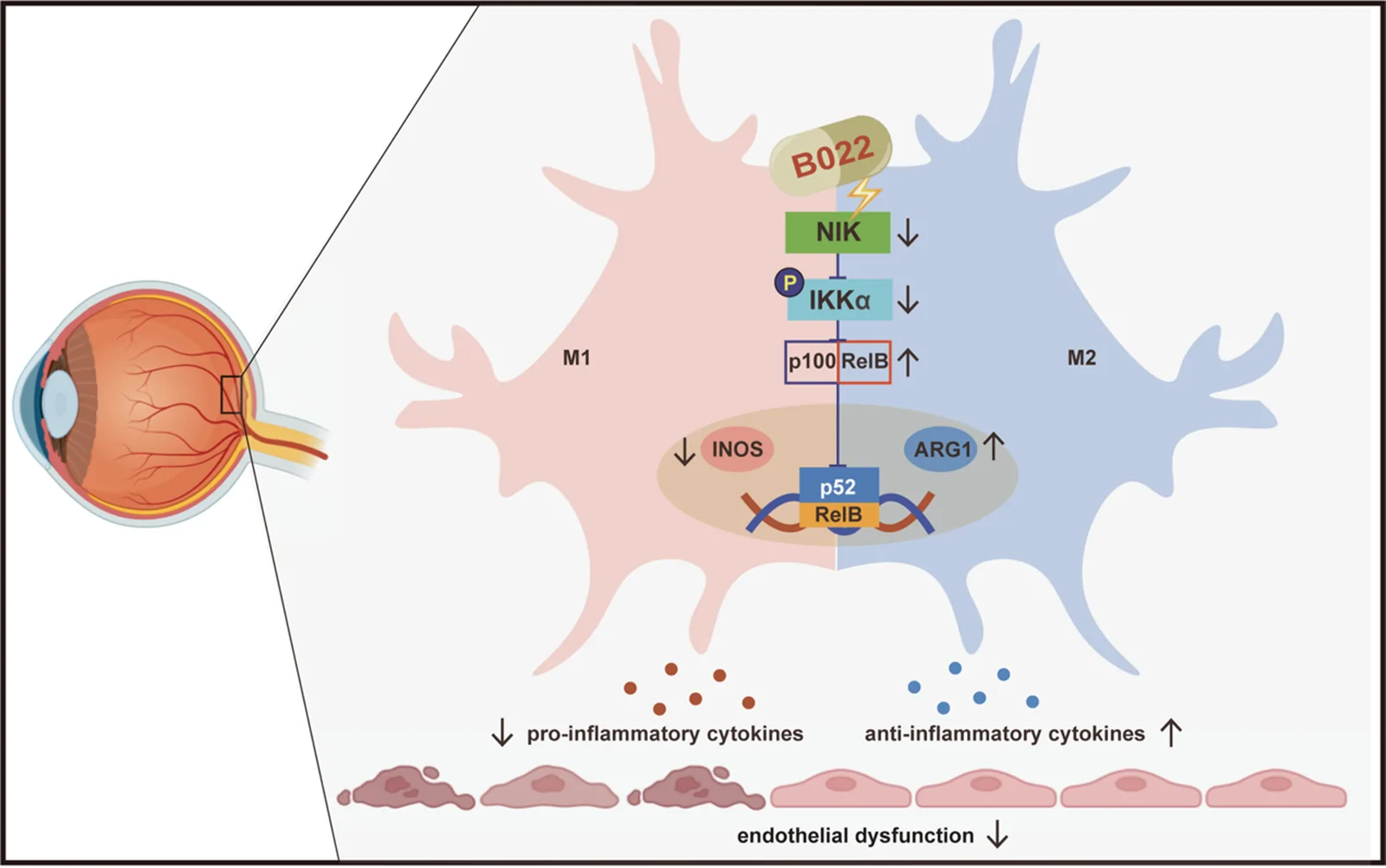
B022 inhibit NIK-mediated microglia polarization in diabetic retinopathy

As described in previous studies, the M1/M2 microglia balance governs the inflammation process, in which control plays a key role in DR. Therefore, clarifying the mechanism of maintenance of M1/M2 balance may provide novel insight for DR. Our study confirmed the role of NIK in the M1/M2 polarization of microglia in the diabetic retina, demonstrating an unknown regulatory mechanism whereby microglia M1/M2 polarization is regulated by NIK-mediated non-canonical NF-κB signaling (Fig. 8).

In conclusion, combined with all findings, we showed that NIK regulated the M1/M2 retinal microglia polarization, which provides a new key molecular target for diabetic retinopathy.

## Supplementary Information

Below is the link to the electronic supplementary material.

Supplementary Material 1

## Data Availability

The data supporting the conclusions of this article are available from the corresponding author upon reasonable request.
