# Supplementary material for: Targeting NIK-mediated microglial polarization: a novel therapeutic strategy for diabetic retinopathy
Source: J Transl Med. 2026 Aug 17;24:1417. doi: 10.1186/s12967-026-08728-4 (PMC13757138; doi:10.1186/s12967-026-08728-4)
Supplement: Supplementary file 1 [file 12967_2026_8728_MOESM1_ESM.docx]

**Figure S1** A. The protein levels of iNOS (M1 marker) and Arg-1 (M2 marker) in mouse retinas were measured by WB. B-C. Relative protein expression of A. D. The expression of NIK in different cell line treated with AGE were detected by WB. E. Retinal microglia were double stained with IBA1(red) and NIK(green). F-G. Relative protein expression of INOS and AGE1 in Figure 3F. H. Mean fluorescence intensity of NFKB2 in Figure 4D. Data are presented as means ± SD. **P* < 0.05, ***P* < 0.01.
